# Supplementary material for: Frequency-selective actuation of liquid crystalline elastomer actuators with radio-frequency
Source: Nat Commun. 2025 Aug 7;16:7292. doi: 10.1038/s41467-025-62313-9 (PMC12332080; doi:10.1038/s41467-025-62313-9)
Supplement: Supplementary file 1 — Supplementary Information [file 41467_2025_62313_MOESM1_ESM.pdf]

Supplementary Information for Frequency-Selective Actuation of Liquid Crystalline Elastomer Actuators with Radio-Frequency

Yiwen Song[⋆,*,1], *Zefang Li*[⋆,2,3], *Mason Zadan*[2,4], *Jingxian Wang*[5], *Swarun Kumar*[*,1], *Carmel Majidi*[*,1,2]

[⋆]*These authors contribute equally to this paper.*

[1] Department of Electrical and Computer Engineering, Carnegie Mellon University
[2] Department of Mechanical Engineering, Carnegie Mellon University
[3] Department of Civil and Systems Engineering, Johns Hopkins University
[4] Koch Institute for Integrative Cancer Research, Massachusetts Institute of Technology
[5] Computer Science Department, National University of Singapore
[*]Corresponding authors: yiwens2@andrew.cmu.edu, swarun@cmu.edu, cmajidi@andrew.cmu.edu

Contents

1 Power Analysis **5**

2 RF Heating Test **7**

3 LED Harvester **9**

4 Energy Harvesting **10**

5 LCE Fabrication **11**

6 Setup of the Actuation system **12**

7 Wireless Channel Estimation **13**

8 Supplementary demos **15**

9 Comparison of soft actuators **16**

10 Comparison of Actuation Methods **17**

List of Figures

S1 Barplot of the heat dessipation rate $k/\Delta x$ fitted from collected power and temperature data. 6
S2 Barplot of heat absorption rate cM fitted from collected power and temperature data. 6
S3 Separate plot of Figure 3D. (a) 35 mm trace length. (b) 45 mm trace length. (c) 55 mm trace width. (d) 65 mm trace width. 7
S4 Separate plot of Figure 3E. (a) 0.44 mm trace width. (b) 0.47 mm trace width. (c) 0.50 mm trace width. 8
S5 Separate plot of Figure 3F. (a) Smooth trace by lowering the 3D printer nozzle. (b) Rough trace by lifting the 3D printer nozzle. 8
S6 PCB and schematic layout of the LED harvester 9
S7 Setup of our wireless actuation system 12
S8 Supplementary demos: (A) Power harvesting enabled by the actuator during actuation; (B) Actuation of a single actuator inside a pipe. 15

List of Tables

S1 Tables of LCE recipe. 11
S2 Comparison of soft actuators 16
S3 A comparison between different actuation methods for soft thermally-driven actuators. Efficiency of laser actuation is calculated from the multiplying the wall-plug efficiency of laser transmitters and the light-to-heat efficiency of the absorbing materials. LoS=Line-of-Sight. NLoS=None-Line-of-Sight. For wireless actuation methods, NLoS only refers to non-metallic blockages, as electromagnetic fields can not penetrate through metallic blockages. 17

1 Power Analysis

The power that is transferred to the soft robot can be categorized into the following types by effect: thermal energy that contributes to the heating of the conductive patterns W_T and electrical energy that is used for energy harvesting W_E. The total energy is thus the combination of these three:

$$W = W_T + W_E \tag{1}$$

We first model the energy that causes the heating of the conductive pattern W_T. Heating the conductive patterns by microwave mainly owes to the surface current that is caused by the changing electric and magnetic field of the magnetic wave, and thus by measuring the current that flows through the conductive patterns we can find out the generated power according to Joule's formula

$$\mathrm{d}W_T = P\,\mathrm{d}t = \hat{I}^2 R\,\mathrm{d}t = \hat{I}^2 \rho l\,\mathrm{d}t \tag{2}$$

where $\hat{I}$ is the average current that flows through the conductive patterns. Specifically, $\hat{I} = I_{\max}/\sqrt{2}$ since we are transmitting a electromagnetic signal with AC voltage $\tilde{V} = V_{\max}\cos(\omega t + \phi)$. ρ is the sheet resistivity (resistance per unit length) of the pattern (Ohm/mm) and l is the length of the conductive pattern. However, it is impossible to directly measure the real-time current that is induced by the microwave signal. Instead, we connect both ends of the conductive pattern to a DC current source and record the heating speed (temperature v.s. time) with different amounts of current. Then we further separate two parts from W_t, which are the temperature raise W_i and the heat dissipated W_d, as follows.

$$\mathrm{d}W_T = \mathrm{d}W_i + \mathrm{d}W_d \tag{3}$$

where we can express W_i with heat capacity

$$\mathrm{d}W_i = cM\,\mathrm{d}T \tag{4}$$

where $\mathrm{d}T$ is a small amount of temperature raise, c is the heat capacity of the conductive pattern, and M is the mass of the conductive pattern. Meanwhile, we can also express W_d by Fourier's law of thermal conduction (), where we have the relationship with heat flux $\mathbf{q}$ and temperature T.

$$\mathbf{q} = -k\nabla T = -k\left(\frac{\mathrm{d}T}{\mathrm{d}x}\mathbf{a}_x + \frac{\mathrm{d}T}{\mathrm{d}y}\mathbf{a}_y + \frac{\mathrm{d}T}{\mathrm{d}z}\mathbf{a}_z\right). \tag{5}$$

where k is the thermal conductivity and T is the temperature. Assume the temperature changes rapidly at the surface from T to T_0 within a small distance Δx, we have

$$\mathbf{q} = -k\nabla T = k\frac{T - T_0}{\Delta x}\mathbf{a}_\mathrm{n}. \tag{6}$$

Therefore since the heat flux only goes outside the conductive materials, we have

$$\mathrm{d}W_d = \left(\oint_S \mathbf{q}\,\mathrm{d}\mathbf{s}\right)\mathrm{d}t = 2k\frac{T - T_0}{\Delta x}(la + lz + az)\,\mathrm{d}t \tag{7}$$

where l, a, z are the length, width, thickness of the patterns, respectively.
To conclude, the heat generated by Joule heating is

$$\mathrm{d}W_T = \hat{I}^2\rho l\,\mathrm{d}t = \mathrm{d}W_i + \mathrm{d}W_d = cM\,\mathrm{d}T + 2\frac{k}{\Delta x}(T - T_0)(la + lz + az)\,\mathrm{d}t \tag{8}$$

Therefore, we use data to fit cM and $\frac{k}{\Delta x}$ by our measurements of $\hat{I}, \rho, l, a, z, T$. The barplots are shown in Figure S1 and Figure S2.

$$cM = 2.78 \times 10^{-3}\ \mathrm{J \cdot K^{-1}}, \frac{k}{\Delta x} = 0.787\ \mathrm{J \cdot K^{-1} \cdot m^{-2} \cdot s^{-1}} \tag{9}$$

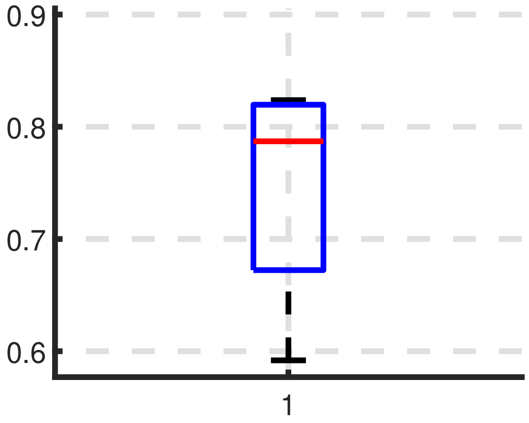

Figure S1: Barplot of the heat dessipation rate $k/\Delta x$ fitted from collected power and temperature data.

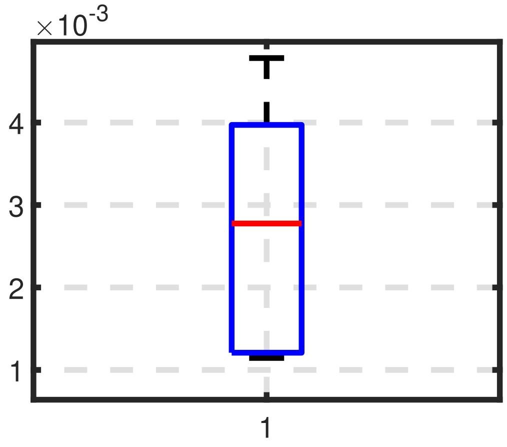

Figure S2: Barplot of heat absorption rate cM fitted from collected power and temperature data.

From our temperature monitor of microwave heating in 30 seconds, we can find that

$$W_T = \int_{T_0}^{T_1} cM\,\mathrm{d}T + 2\int_{t_0}^{t_1} \frac{k}{\Delta x}(T - T_0)(la + lz + az)\,\mathrm{d}t = 5.83\ \mathrm{J} \tag{10}$$

which yields us the average power $\hat{P}_t$ as

$$\hat{P}_t = \frac{W_t}{t_1 - t_0} = 194\ \mathrm{mW} \tag{11}$$

2 RF Heating Test

Separate plots for different radio-frequency heating of conductive traces is demonstrated in this section.

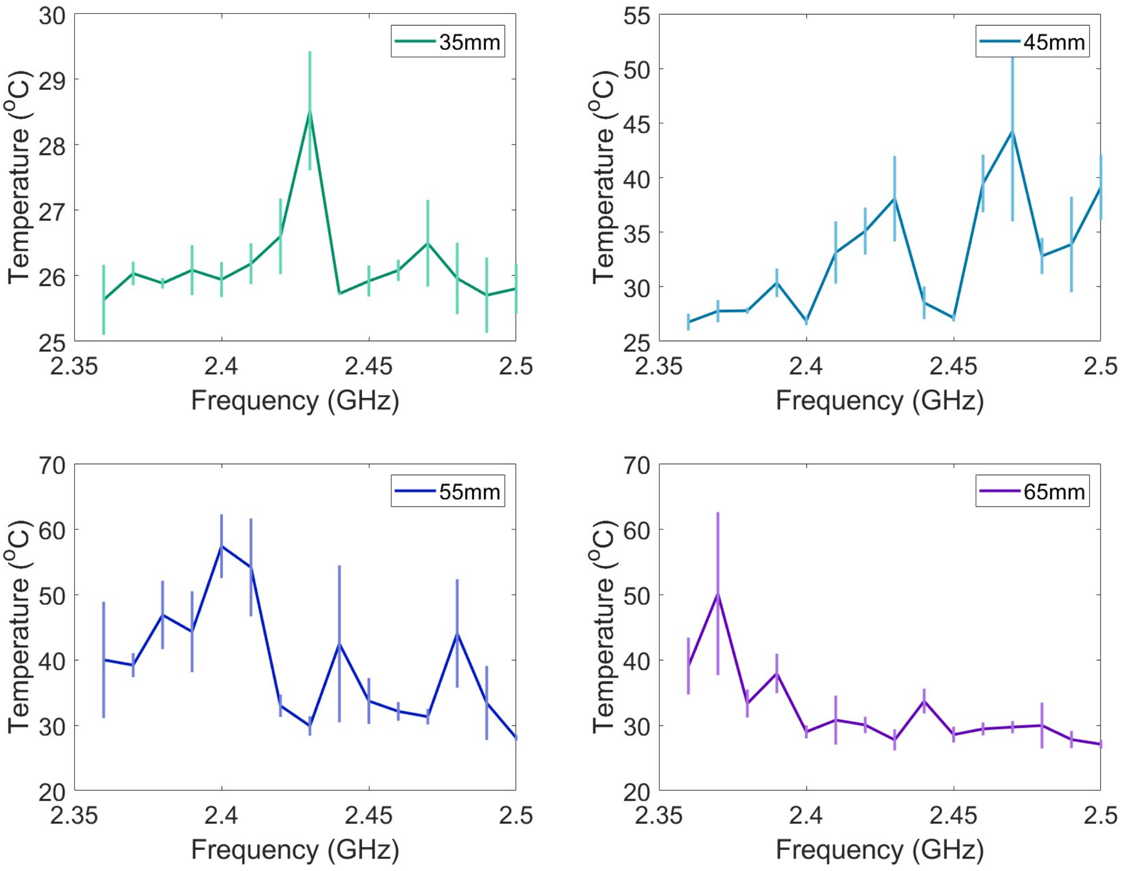

Figure S3: Separate plot of Figure 3D. (a) 35 mm trace length. (b) 45 mm trace length. (c) 55 mm trace width. (d) 65 mm trace width.

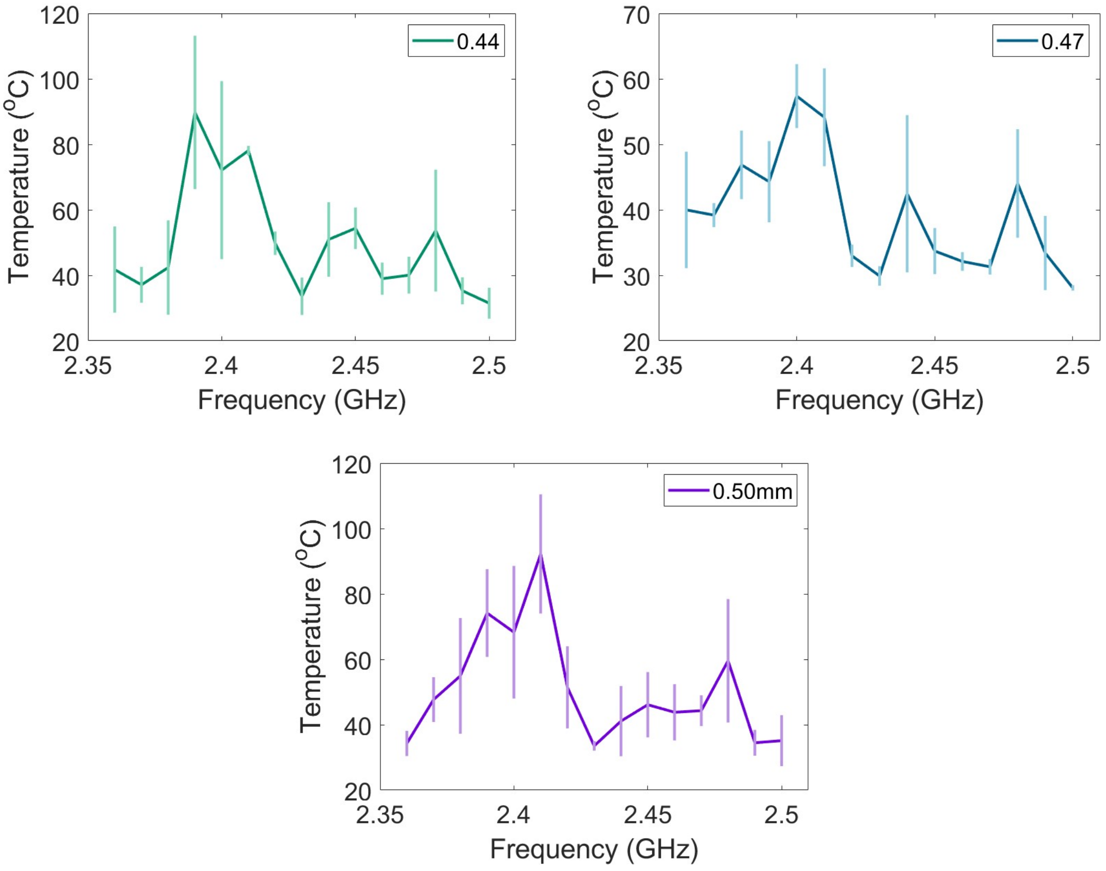

Figure S4: Separate plot of Figure 3E. (a) 0.44 mm trace width. (b) 0.47 mm trace width. (c) 0.50 mm trace width.

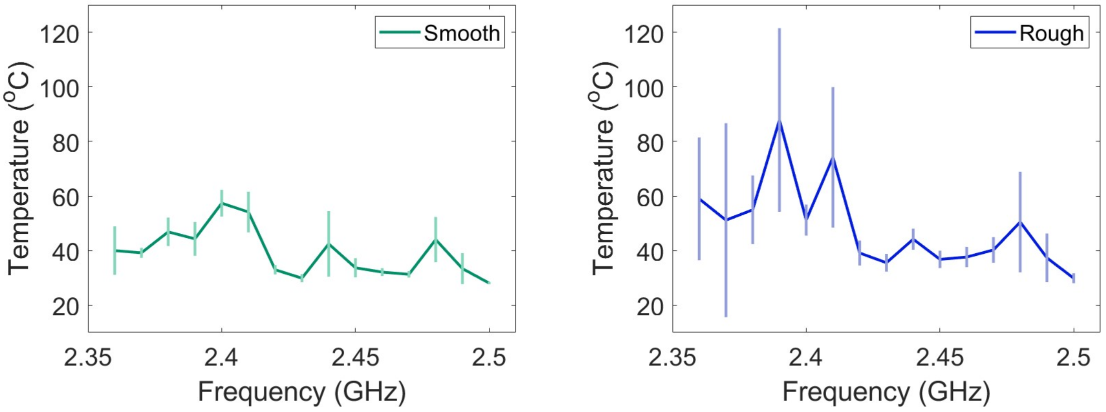

Figure S5: Separate plot of Figure 3F. (a) Smooth trace by lowering the 3D printer nozzle. (b) Rough trace by lifting the 3D printer nozzle.

3 LED Harvester

The schematic and the PCB layout of the LED harvester are shown in Figure S6.

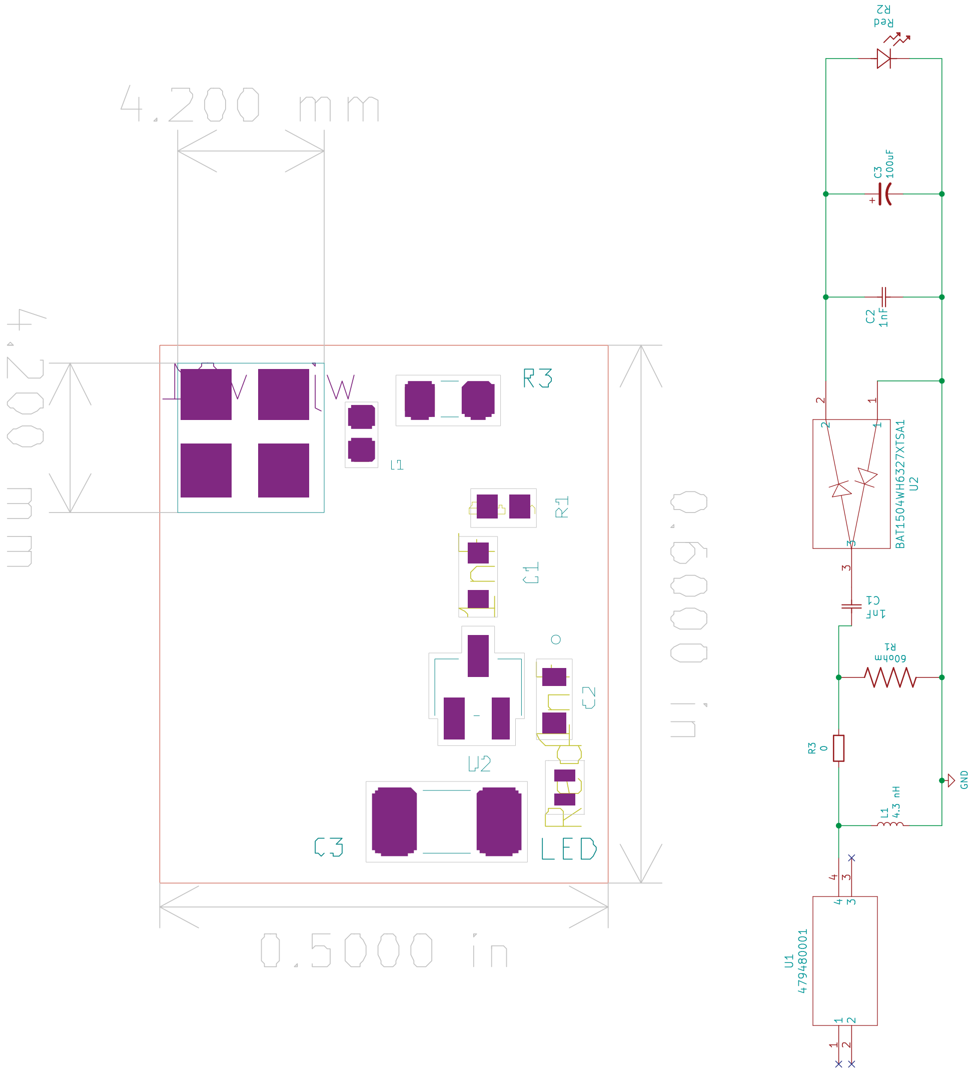

Figure S6: PCB and schematic layout of the LED harvester

4 Energy Harvesting

We use the lightness of custom-designed LED harvesters to characterize the energy harvesting efficiency. Note that the LED can be replaced with other electrical components such as ultrasonic sensors, wireless transceivers, humidity sensors, etc., for different applications. We use a 2.4 GHz spiral antenna to harvest the radio frequency power and use a rectifier and matching circuit to acquire a DC power input for an LED harvester. The LED has a DC-brightness response according to datasheet (`https://fscdn.rohm.com/en/products/databook/datasheet/opto/led/chip_mono/sml-p11-e.pdf`). We observe the brightness obtained in a camera with regard to input power and use linear regression to fit the points. By regression we can find that the overall power that can be harvested by the energy harvesting circuit is 400 mW at 60 W total transmission power.

Therefore, in conclusion, the total power is approximately

$$\hat{P} = \hat{P}_T + \hat{P}_E = 594 \, \mathrm{mW} \tag{12}$$

5 LCE Fabrication

Table S1 shows the table of LCE recipes.

5% Bisphenol molar fraction			10% Bisphenol molar fraction			15% Bisphenol molar fraction		
**Ingredient**	**Weight (g)**	**Volume (mL)**	**Ingredient**	**Weight (g)**	**Volume (mL)**	**Ingredient**	**Weight (g)**	**Volume (mL)**
RM257	2	SOLID	RM257	2	SOLID	RM257	2	SOLID
Bisphenol-512	0.091563	0.080318	Bisphenol-512	0.193299	0.169561	Bisphenol-512	0.307004	0.269302
EDDET	0.526512	0.4701	EDDET	0.555762	0.496216	EDDET	0.588454	0.525405
PETMP	0.124529	0.097288	PETMP	0.131447	0.102693	PETMP	0.139179	0.108734
HHMP	0.002406	SOLID	HHMP	0.00254	SOLID	HHMP	0.002689	SOLID
TEA	0.206296	0.284155	TEA	0.217757	0.299941	TEA	0.230566	0.317584
Toluene	0.652517	0.752615	Toluene	0.688768	0.794427	Toluene	0.729284	0.841158

Table S1: Tables of LCE recipe.

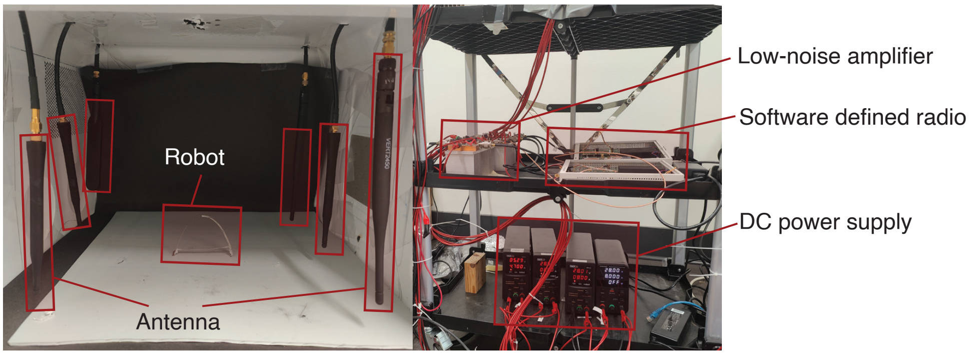

Figure S7: Setup of our wireless actuation system

6 Setup of the Actuation system

Figure S7 shows a photo of our setup for the actuation platform. The antennas and the robot are inside an enclosure to prevent power leakage in order to comply with FCC regulations. The signal generator is composed of low-noise amplifiers (one for each antenna), a software-defined radio (one RF chain for each antenna), and DC power supplies for the amplifiers.

7 Wireless Channel Estimation

Wireless channel estimation consists of two parts: channel estimation and channel interpolation. Since the wireless channel from each antenna is unknown inside the working environment of the soft robot, we first place multiple LED harvesters equipped with a 2.45 GHz antenna. The power received by the LED harvesters can be calculated through observing the brightness of the LEDs.

Specifically, we design a channel probing algorithm to probe the LED harvesters using low-power 2.4 GHz signals. The challenge however is that the brightness of LEDs only shows the amplitude of the channel. In other words, we cannot directly measure the phase of the wireless channel by the brightness of LEDs. RFact estimates the wireless channel at each location that these energy harvesters are deployed The channel information can be measured by the brightness of the LED equipped on the energy harvesters. by applying various sets of beamforming vectors across all antennas, and measuring the brightness of the LED array. Specifically, we design a novel closed-loop probing algorithm using low-power 2.45 GHz signals to probe the LEDs at desired locations. The challenge however is that the brightness of LEDs only contains the amplitude of the channel. In other words, we cannot directly measure the phase of the wireless channel by the brightness of LEDs. The challenge however is that the brightness of LEDs can only contain the amplitude $\mathbf{b}_k$ of the wireless channel $\left|\mathbf{W}_k^\top \mathbf{h}\right|$. In other words, we can not get the phase information of the wireless channel only relying on the brightness of LED arrays. Thus, RFact must solve the *phase retrieval* problem. At a high level, RFact solves it by probing the LED harvesters with multiple beamforming vectors $\mathbf{W}_k$ where k represents the k-th probe. RFact finds the wireless channels of LED harvesters $\mathbf{h}$ by solving:

$$\min_{\mathbf{h}} \quad \left|\left|\left|\mathbf{W}^\top \mathbf{h}\right| - \mathbf{b}\right|\right|_2^2 \tag{13}$$

where $\mathbf{W} = [\mathbf{W}_1, \mathbf{W}_2, \cdots, \mathbf{W}_K]$, and $\mathbf{b}_1, \mathbf{b}_2, \cdots, \mathbf{b}_K \in \mathbb{R}$ are measured amplitude of the LED harvesters for each probe. We adapt PhaseLift (*1*) to retrieve the phase and solve Eqn. 13. The algorithm (*1*) is guaranteed to converge when we apply $4 \times N$ times of channel probes, where N is the number of antennas. In our experiments, we deploy 4 antennas and apply 16 channel probing to infer the LED array's wireless channels. The computation latency to solve Eqn. 13 takes less than 1 second.

Thus, RFact uses $4 \times N$ to guarantee the convergence of its algorithm. The actual probing signals (precoders) are randomly generated complex Gaussian vectors. The probing results are normalized to have a mean of one to meet the requirement of the PhaseLift algorithm. Normalizing the probing results as well as the wireless channel $\mathbf{h}$ does not matter in our experiments, since we only want to infer the relative strength and phase difference of different elements in $\mathbf{h}$ for beamforming, rather than absolute values.

Interpolating channel information spatially: In compliance with FCC regulations, our high-power beamforming actuation is performed inside an RF shielded space (e.g., a Faraday sleeve) to prevent microwave leakage. The shielded space can be seen as a cavity resonator where EM signals form standing waves. Thus, the x, y, z partitions of the electric field and magnetic field can be represented by a sinusoid product of the coordinates. We then use the Poynting's theorem to model the power distribution inside the cavity resonator with a combination of sinusoidal waves. We can compute the power density $S = |\mathbf{S}| = |\mathbf{E} \times \mathbf{H}|$ as follows:

$$\begin{aligned} S(x,y,z) &= \left|\mathbf{E}(x,y,z) \times \mathbf{H}(x,y,z)\right| = \frac{1}{2}\epsilon\left|\mathbf{E}(x,y,z)\right|^2 \\ &= C^2 \left[\sin^2\left(\frac{\pi x}{a}\right)\sin^2\left(\frac{\pi y}{b}\right)\sin^2\left(\frac{\pi z}{l}\right)\right] \end{aligned} \tag{14}$$

where C, a, b, l are all constants. We then leverage the wireless channel $\mathbf{h}$ at each LED harvester's location (x_p, y_p, z_p) to solve the following optimization problem and interpolate the channels at the remaining locations:

$$\min_{C_n, a_n, b_n, l_n} \quad \sum_p \Big| S(x_p, y_p, z_p) - \big|\mathbf{h}_n(x_p, y_p, z_p)\big| \Big|^2 \tag{15}$$

this optimization is locally convex and can be solved by gradient descent. The interpolated channel of the antenna n at the desired position (x, y, z) would be $\Big|\hat{\mathbf{h}}_n(x, y, z)\Big| = S(x, y, z)$.

Interpolating channel information in frequency domain: The constants of the standing wave a, b, l mentioned in the above equation are wavelength-dependent. Imaging a standing wave formed between two ideal reflectors, the standing wave formed at the two reflectors can be expressed as

$$S(x) = C^2 \sin^2\left(2\pi \frac{d}{\lambda}\right), \tag{16}$$

where $\lambda = c/f$. Therefore, the phase in the $\sin^2$ term increases with regard to the frequency f. In such a way, we can interpolate the phase of the field strength at the same point with regard to different frequencies by a linear interpolation. Meanwhile, assume an large plane where the standing wave is formed, and the wave source is fixed, then the wavelength of the standing wave described in Eq. 16 decreases linearly with the wavelength λ. Therefore, we can interpolate the signal strength of a single point with regard to different frequencies by a sinusoidal interpolation.

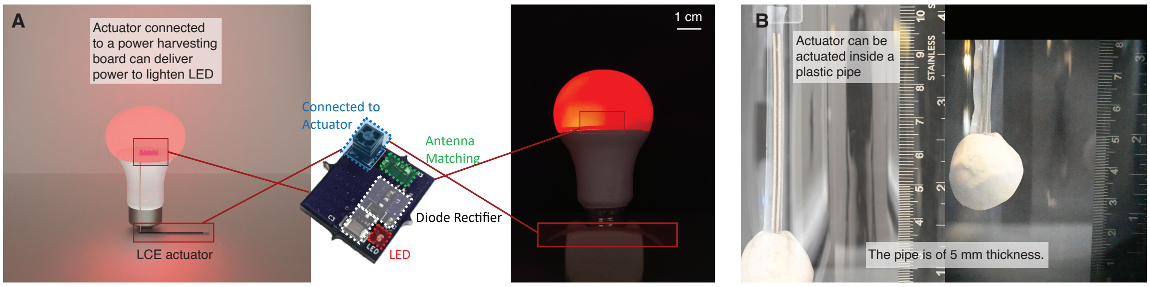

Figure S8: Supplementary demos: (A) Power harvesting enabled by the actuator during actuation; (B) Actuation of a single actuator inside a pipe.

8 Supplementary demos

We show three supplementary demos for an extra explanation of the capability of RFact.

Wireless Power Harvesting with the Actuator: The radio-frequency powers the actuation of the soft robot by generating inductive heat inside the conductive patterns in the actuator. This demonstration proposes a more efficient usage of the captured power, which is using the induced current not only for actuation, but also for powering electronics. The underlying idea is that the actuator can not only serve as a mechanical part that drive the motion of the soft robot, but also serve as an antenna that harvests the RF power to serve the low-power on-board electronics, such as ultrasonic sensors, temperature sensors, etc. The video in Supplementary Movie 3 shows an LED harvester, connected to the actuator that serve as an antenna, is generally lighten up when we gradually tune the output power from 0 W to 5 W. In Supplementary Information Section 1, we calculate that around 400 mW of power can be generated by one trace to power an attached device, when the total output power is set to a maximum of 60 W.

Actuation under occlusion: Wireless actuation has the capability of actuating soft robots under non-metallic occlusion such as plastic pipes and walls. Supplementary Movie 4 shows the actuator under occlusion. RFact demonstrates that the actuation of actuators is slightly affected by occlusion. With a 1.5 cm-thick pipe covering the actuator in all directions, the time for the same actuator to fully actuate extends from 13 sec to 15 sec (Supplementary Movie 4).

Soft Actuator	Method of Actuation	Actuation strain	Actuation stress
LCE, 5% Bisphenol-512	Heat, above 50°C.	30%	350 kPa
SMA, Ni-Ti spring (*2*)	Heat, above 120°C.	8%	1 MPa
Dielectric elastomer, DE stack (*3*)	Voltage, above 4 kV.	30%	50 kPa
Pneumatic, McKibben (*4*)	Air pump, above 10W.	30%	3 MPa
Ionic diffusion, IPCNC (*5*)	Voltage, above 4V.	8%	8 kPa

Table S2: Comparison of soft actuators

9 Comparison of soft actuators

Table S2 shows the trade-off between five different types of soft actuators. LCE and SMA are actuated by heat. Compared to dielectric elastomer, pneumatic muscles, and inoic diffusion actuators, they are easier to be actuated remotely from a wireless source of heat or power transfer. Compared to SMA springs, LCE provides higher strain but lower stress at a much lower actuation temperature. Dielectric elastomers are actuated at a faster rate, but require high voltage and provide small stress. Pneumatic actuators provide high stress and strain, but require large power input and a tethered air tube. Ionic diffusion actuators can be actuated with a small voltage and power, but produce smaller strain and stress.

**Actuation Method**	**Power Efficiency (%) From electric to heat**	**Spatial Selectivity**	**Frequency Selectivity**	**Range of Operation**	**Bulkiness**
Wired (*6*)	∼20	Very good	No	Wire length (NLoS)	High (wire tethering)
Batteries (*6*)	∼20	Very good	No	N/A (NLoS)	High (battery weights)
Laser (*7*)	∼30*	Good	Yes	∼10 cm (LoS)	Low
Near-field coupling (*8*)	∼30	Bad	No	<10 cm (NLoS)	Low
RF (ours)	∼20	Normal	Yes	∼30 cm (NLoS)	Low

Table S3: A comparison between different actuation methods for soft thermally-driven actuators. Efficiency of laser actuation is calculated from the multiplying the wall-plug efficiency of laser transmitters and the light-to-heat efficiency of the absorbing materials. LoS=Line-of-Sight. NLoS=None-Line-of-Sight. For wireless actuation methods, NLoS only refers to non-metallic blockages, as electromagnetic fields can not penetrate through metallic blockages.

10 Comparison of Actuation Methods

We compare RFact with existing actuation methods for soft thermally-driven actuators. Currently, the maximum power efficiency that can be achieved by different actuation systems remains similar. The main challenge in improving the power efficiency for such system is designing more efficient actuators to convert thermal energy into mechanical energy. The spatial selectivity of our system is worse than wired/battery/laser actuation, but when combining spatial selectivity with frequency selectivity, RFact can achieve selective actuation of different actuators that are very nearby. Compared to other wireless actuation methods, RFact achieves non-line-of-sight actuation at a larger range. Compared to wired and battery actuation, wireless actuation has the advantage in low bulkiness and mobility.

References

1. E. J. Candes, T. Strohmer, V. Voroninski, *Communications on Pure and Applied Mathematics* **66**, 1241 (2013).
2. M.-S. Kim, *et al.*, *Advanced Materials* **35**, 2208517 (2023).
3. G. Kovacs, L. Düring, S. Michel, G. Terrasi, *Sensors and actuators A: Physical* **155**, 299 (2009).
4. B. Tondu, P. Lopez, *IEEE control systems Magazine* **20**, 15 (2000).
5. S. Liu, *et al.*, *Advanced functional materials* **20**, 3266 (2010).
6. M. Zadan, *et al.*, *Advanced Materials* p. 2200857 (2022).
7. K. Gu, H. Zhong, *Light: Science & Applications* **12**, 120 (2023).
8. M.-S. Huang, Y.-L. Huang, *International Journal of Heat and Mass Transfer* **53**, 2414 (2010).
