# Supplementary material for: Frequency-selective actuation of liquid crystalline elastomer actuators with radio-frequency
Source: Nat Commun. 2025 Aug 7;16:7292. doi: 10.1038/s41467-025-62313-9 (PMC12332080; doi:10.1038/s41467-025-62313-9)
Supplement: Supplementary file 2 — Description of Additonal Supplementary Files [file 41467_2025_62313_MOESM2_ESM.pdf]

Description of Additional Supplementary Files

Supplementary Movie 1: Video demonstrating the wireless actuation of a single actuator with a energy storage and release mechanism.

Supplementary Movie 2: Video demonstrating the selective wireless actuation and the capability of using selective wireless actuation to enable a walking robot.

Supplementary Movie 3: Video demonstrating the power harvesting capability enabled by connecting a power harvesting circuit to the actuator, and using the power harvesting capability to power an LED.

Supplementary Movie 4: Video demonstrating the wireless system actuating an actuator with a load of 0.5 N under plastic occlusion.

Supplementary Movie 5: Video demonstrating the wireless system is capable of transmitting signal selectively to two actuators and using a power harvesting circuit to capture the signal.

Supplementary Movie 6: Video demonstrating a crawling robot working inside an enclosed pipe, collecting object inside the pipe, by using selective actuation in non-line-of-sight.
